# Exploring the application of large language models in coding the experiencing scale (EXP)

**DOI:** 10.1080/28324765.2026.2664163

**Published:** 2026-04-23

**Authors:** Brian Yim, J. Christopher Muran, Qianying Ren, Bernard Gorman

**Affiliations:** a Gordon F. Derner School of Psychology, Adelphi University, New York, USA; b Department of Computer Science and Engineering, School of Computing, University of Connecticut, Storrs, Connecticut

**Keywords:** The experiencing scale, large language models, chatgpt, psychotherapy process coding, prompt engineering

## Abstract

Psychotherapy process measures like the Experiencing Scale (EXP) offer valuable insight into clinical interactions but are time-intensive to code. Large language models (LLMs) like ChatGPT have the potential to streamline this process, but empirical validation is nascent. This exploratory study aimed to provide a proof-of-concept coding the EXP using ChatGPT with special attention to ethical considerations, limitations, and future directions. ChatGPT was used to code 79 psychotherapy transcripts drawn from the EXP manual. Multiple models of ChatGPT were tested using varied few-shot learning prompt engineering protocols. Data collection occurred in three phases, during which models rated both modal and peak EXP scores for all transcripts. ChatGPT demonstrated moderate agreement with manual reference ratings. An efficient configuration (o3-mini, 5-shot prompting) yielded moderate reliability for both modal EXP scores (ICC[3,1] = .67, 95% CI [.53, .79]) and peak EXP scores (ICC[3,1] = .71, 95% CI [.58, .81]). LLMs may feasibly augment or replace human EXP coders under certain conditions. However, evidence is preliminary and ethical and technical limitations remain. Future research should validate the present methodology using out-of-manual data, assess potential pretraining exposure, and explore locally hosted LLM applications to mitigate privacy concerns.

Large language models (LLMs) are an emerging technology that have rapidly advanced in recent years. Unlike traditional computational methods of the past, these models are not explicitly programmed for specific tasks; instead, they rely on predictive modelling derived from extensive training on large and diverse datasets. These datasets encompass a broad range of topics, including specialised domains such as psychotherapy and psychological research, thereby allowing these models to approximate aspects of these concepts. Through extensive training data, LLMs such as ChatGPT show strong performance across many language tasks; however, their logical reasoning remains inconsistent, particularly in out-of-distribution settings and certain inference benchmarks (Gupta et al., 2023; Liu et al., 2023). These capabilities—including text analysis, semantic understanding, and pattern recognition—are highly relevant to psychotherapy process research.

Exploratory research in the use of LLMs has begun spanning across domains, including medicine (Wu et al., 2024), higher education (Raitskaya & Lambovska, 2024), mental health (Hua et al., 2025), psychoeducation (Maurya et al., 2025), and psychotherapy applications (Alanzi et al., 2024). Within psychotherapy *process* research, researchers have demonstrated that LLMs can automatically rate patient engagement with strong psychometric properties (Eberhardt et al., 2025) and classify therapist microcounseling skills with good accuracy and interrater reliability (Hammerfald et al., 2025). These findings suggest that LLM-based approaches may provide scalable, automated, and interpretable alternatives to labour-intensive manual coding.

ChatGPT, one of the most widely used and high-performing LLMs (AlpacaEval, 2025), may serve as a reasonable proof-of-concept for this work. A key feature of ChatGPT, like many other LLMs, is its capacity for few-shot learning—the ability to generalise from a handful of examples (commonly 3–10) provided within a prompt—which may enable efficient task performance without requiring large annotated datasets (Brown et al., 2020). This property is especially advantageous for psychotherapy research, where training datasets are often limited.

No prior research has applied LLMs to the Experiencing Scale (EXP; Gendlin, 1962), a measure of in-session emotional processing. The present study addresses this gap by exploring a proof-of-concept methodology for automating EXP coding using ChatGPT. Given their accessibility, relatively low cost, and rapidly improving performance, LLMs represent a promising tool for advancing scalable, reproducible, and efficient psychotherapy process research.

## Ethical and practical considerations

Although LLMs such as ChatGPT hold potential for advancing psychotherapy research, they also raise serious concerns about privacy, bias, and confidentiality. ChatGPT is a proprietary, cloud-based tool, meaning that data are transmitted to external servers where they may be stored for up to 30 days and disclosed under certain conditions (e.g. legal requests, third-party processing; OpenAI, 2024b). Moreover, psychotherapy transcripts are highly sensitive, and even when de-identified, researchers argue that there remains a risk of re-identification, particularly when combined with other datasets or contextual information (Wang et al., 2023).

Another key challenge is model reliability and bias. LLMs are prone to ‘hallucinations’—producing fluent but factually incorrect outputs (Xu et al., 2024). The ‘black box’ nature of LLM amplifies this effect, as their internal decision-making processes are largely opaque. Researchers can observe the input and output but cannot easily trace why a model produced a particular rating or classification. Without clear interpretability, automated ratings risk compromising accountability, representing a persistent limitation of all LLM-based research.

Despite these risks, emerging work suggests that, rather than halting research in this area, a more productive approach may be to proceed cautiously under appropriate ethical safeguards. For example, some studies have sought to mitigate privacy concerns by using locally deployed LLMs to automate patient engagement ratings with real-world data while maintaining confidentiality (Eberhardt et al., 2025). There is also some evidence that hallucination rates have declined in more recent model iterations (OpenAI, 2024a), although findings vary depending on task and evaluation context (Parker et al., 2024). Taken together, these considerations highlight the importance of developing approaches that balance innovation with the protection of participant privacy and scientific integrity. These issues are considered further in the Discussion.

The present study is presented as a proof-of-concept, using ChatGPT and publicly available EXP manual transcripts as an example application of LLM-based coding. The methodology is intended as a model that can be applied to other LLMs, including open-source and locally deployed systems, which may be preferable when working with sensitive real-world transcripts. All data contained no protected patient information.

## The experiencing scale

The Experiencing Scale is a psychotherapy process measure developed by Eugene Gendlin (1962) and colleagues (Klein et al., 1986, 1969) to assess the depth of a client’s emotional processing and meaning-making, or experiencing, during psychotherapy sessions. It is informed by the humanistic tradition and has been considered a gold standard in psychotherapy process research for evaluating in-session client engagement (Pascual-Leone et al., 2016). The EXP was selected for this study for three reasons: it can be reliably coded from transcripts, it has established utility in psychotherapy research, and its sensitivity to affective depth and reflective awareness makes it well-suited for identifying moments of rupture and repair—a central focus of our Alliance-Focused Training research programme (AFT; Muran & Eubanks, 2020; Safran & Muran, 2000).

The Experiencing Scale is a 7-point observational measure used to assess a client’s depth of experiencing based on audio or transcript data. Coders are trained to rate a session for the modal (most common level) and peak (highest level) scores. On the lower end of the scale, clients are described as ‘impersonal’ or ‘detached,’ while at the highest end of the scale, client experiencing is described as ‘expansive’ or ‘illuminating.’

Prior outcome research has supported the finding that higher EXP scores are associated with better psychotherapy outcomes (Elliott et al., 2013; Hendricks, 2002). Building upon these findings and examining the broader literature, Pascual-Leone and Yeryomenko (2017) conducted a meta-analysis and found that EXP scores predicted treatment outcomes, with a small to medium association between EXP scores and self-reported outcomes (r = –.19).

## Human process-coding challenges

Human process coding has long played an important role in psychotherapy research and has also been used as a training tool for clinicians, supporting the development of clinical judgement and therapeutic skills (Westra & Di Bartolomeo, 2024). However, human coding also has limitations. Traditional coding of psychotherapy measures such as the EXP can achieve strong interrater reliability when conducted by trained experts. However, this level of reliability requires extensive training and is both time- and labour-intensive: coders typically require upwards of 20 hours of training to reach reliability, and a single 50-minute session can take approximately two hours to code (Klein et al., 1969), thereby constraining studies to relatively small sample sizes. Even after training, human coders as instruments remain susceptible to effects such as observer drift, subjective bias, and fatigue effects (Girard & Cohn, 2016).

## Objective

The present study aims to develop a proof-of-concept, reliable, automated method for coding the EXP using ChatGPT, drawing on examples from the EXP manual. Its goal is to evaluate feasibility, establish baseline agreement with manual reference ratings, and identify potential risks and limitations. Automating this process could significantly enhance the efficiency of psychotherapy process research and lay the groundwork for further research in this domain. Special attention is given to the practical and ethical considerations of using ChatGPT in psychotherapy research.

## Method

### Dataset

The dataset comprised 79 transcripts sourced from the EXP Manual (Klein et al., 1969). An undergraduate research assistant digitised each transcript, segmenting and cleaning each by removing all non-patient (i.e. therapist) speech. The resulting files included only patient utterances and were capped at 3,000 words per file to comply with ChatGPT’s input limitations (based on a ~4,096-token limit at the time of data cleaning, Q3 2024; OpenAI, n.d.-a). No transcripts were truncated due to the segmentation of files.

### Measures and variables

The primary measure used was the EXP (Gendlin, 1962), a process measure designed to assess the depth of a client’s emotional experiencing in psychotherapy sessions. The EXP scores range from 1 (low experiencing) to 7 (high experiencing), and are assigned based on the content and quality of client utterances. Stage 1 is described as impersonal or externally focused content, whereas Stage 7 described as confident, integrated emotional insight.

The primary variables were the mode and peak EXP scores assigned for each transcript, rated independently by ChatGPT and expert human coders. Different ChatGPT models and prompt protocols acted as separate exposure conditions. No additional covariates were considered, as ratings were based solely on example transcript text and no systematic differences across transcripts were expected to bias results. All ratings were based on utterances compiled at the transcript level, consistent with EXP conventions. Ratings were based solely on transcript text.

### Reliability analysis

The primary psychometric used was ICC(3,1), which represents a two-way mixed-effects model assessing absolute agreement for single measurements. The ICC(3,1) model was selected because the study involved comparing ChatGPT-generated scores against expert-rated scores, with two raters evaluating the same set of transcripts. Separate ICCs were calculated for both mode and peak EXP scores across all transcripts, protocols, and testing phases.

As a secondary metric of scoring accuracy, mean absolute differences (MAD) between ChatGPT and expert scores were calculated by subtracting expert-rated scores from ChatGPT-rated scores, taking the absolute values, and averaging them out across the transcripts. We also evaluated practical feasibility, including cost-effectiveness and implementation considerations (e.g. token usage and processing time), such that when performance metrics were comparable, protocols with fewer exemplars were preferred. ICCs for all phases were computed in SPSS, from which F statistics and degrees of freedom are reported. To avoid overlap between prompting and evaluation, any transcript used as a few-shot example in a given protocol were excluded from both ICC and MAD calculations for the corresponding protocol.

The sample size for this study was determined by the number of available transcripts (*N* = 79) included in the EXP manual. Post-hoc power analysis revealed that, based on guidelines from Research Design in Clinical Psychology (Kazdin, 2003), the study provided adequate power (>.99) to detect ICCs at the. 75 threshold (i.e. the minimum for research utility).

### Models and prompting protocols

ChatGPT was selected due to its strong instruction-following performance on AlpacaEval and its widespread availability at the time of testing (AlpacaEval, 2025). As the study relied exclusively on EXP manual transcripts, privacy concerns were not implicated; thus, ChatGPT was an appropriate choice for this proof-of-concept investigation. Multiple versions of the ChatGPT model were tested throughout the study, including GPT-4o and GPT 4.5 (flagship models), and o3-mini and o3-mini-high (reasoning models). These four represented the primary options available at the time of testing and were selected to enable comparison broadly across different models. While OpenAI’s flagship models prioritise speed and general-purpose capabilities, their reasoning models are designed for complex, multi-step problem-solving, and are more resource-intensive. All ChatGPT testing data was collected in Q1 of 2025.

The prompting protocol was developed by the first author (a PhD candidate in clinical psychology with a BASc in computer engineering) in collaboration with the third author, a senior doctoral candidate in computer science. At the time of methodological development (Q4 2024), formalised best-practice guidance for prompt engineering in specialised, instruction-driven coding tasks was limited; accordingly, prompt construction followed an iterative process grounded in the EXP manual, few-shot learning (Brown et al., 2020), and chain-of-thought principles (Wei et al., 2022). All prompts were developed with the assistance of GPT-4o to improve clarity and consistency of instruction phrasing, while preserving the content and decision rules specified in the manual. This process was used to state coding guidelines in a format more readily interpretable by LLMs, rather than to iteratively optimise prompts based on model performance.

The project involved three phases of data collection, each using prompts specifically engineered for ChatGPT to interpret. These phases included an initial testing phase, a model comparison phase, and a protocol testing phase. We will first describe the prompts developed and used, followed by describing the three phases of data collection.

#### Prompt engineering

Prompt engineering was a central component of this study. Given that language models function as few-shot learners (Brown et al., 2020), the prompting protocols were intentionally designed to test 0-shot to 10-shot learning (i.e. the models were given between 0 to 10 examples). This range was also chosen for its practical feasibility, as it strikes a reasonable balance between providing sufficient context to improve performance while avoiding overly long prompts that can degrade model performance and increase error or hallucination rates due to limitations in how large language models attend to and utilise long contexts (​​​​​Liu et al., 2023). Three structured prompt sets were used to guide ChatGPT’s scoring of the EXP.

1. **Scale Definition Prompt**

The first prompt introduced the EXP scale definitions to provide a foundational reference for ChatGPT. This supplied the model with the full 7-point EXP scale, from detached external descriptions (Stage 1) to fully integrated self-reflection (Stage 7). The following is an excerpt from the prompt used, with the complete text available in the Supplemental Online Materials - Scale Definition Prompt:

“Use the following reference to identify and score statements or segments on the EXP (Experiencing) scale. Match the content to the highest applicable stage, based on the depth of self-reference, emotional awareness, and internal processing.

Stage 1—Impersonal, Detached

Key traits: No personal involvement; speaker is a passive observer or gives generic commentary…”

2. **Few-Shot Example Prompts**

The second set of prompts provided example transcript segments with expert-assigned EXP mode and peak scores, alongside reasoning. These were presented over several consecutive messages to emulate few-shot learning. The following is an excerpt from the prompt used, with the complete text available in the Supplemental Online Materials - Few-Shot Example Prompts:

“Here are a few example transcript segments, with their corresponding EXP Mode, Peak, and Explanation. Use this structure and reasoning style when scoring future segments. I will provide seven therapy transcripts with their assigned EXP scores and reasoning in seven separate messages. Here is the first one:

Segment B-5, Mode EXP: 1, Peak EXP: 2

S: In so far as how she's been to my brother, now ah he, it's okay with him if she has a cat, so I ah arranged to buy ah a Siamese kitten down here for $20….”

3. **Coding Instruction Prompt**

The third set of prompts instructed ChatGPT to take on the role of an expert rater and apply the EXP scale to the dataset of 79 transcripts, rating several transcript segments per prompt. The model was reminded of the previously provided scale definitions and example reasoning structure before being asked to rate several transcript segments. The following is an excerpt from the prompt used, with the complete text available in the Supplemental Online Materials - Coding Instruction Prompt:

“You are an expert trained in applying the EXP (Experiencing) Scale, which ranges from 1 (least personal/impersonal) to 7 (highest emotional insight/self-awareness). The detailed EXP scale definitions and representative examples have already been provided in a previous message. Refer explicitly to those guidelines when analysing content. Your task is to analyse segments of a therapy transcript. Each segment comprises multiple utterances (turns of speech). For each segment, complete the following steps:

1. Carefully assess and assign an EXP score (1 to 7) to each individual utterance using the provided EXP manual and stage descriptions. Many short utterances such as ‘OK,’ ‘uhm,’ ‘Yes sir,’ ‘well…,’ or ‘No,’ do not count—skip those…”

### Procedure

All inferences were conducted via the ChatGPT application (macOS) under a ChatGPT Plus subscription during specified testing windows (see Reproducibility Checklist in Supplemental Online Materials for exact dates, models and settings). Because the web interface abstracts inference parameters and may change routing, system prompts, or model behaviour over time, results should be interpreted as reflecting the ChatGPT product experience at the time of testing rather than as fixed properties of a static model configuration. ChatGPT settings were configured to disable data sharing with OpenAI to the extent permitted by the platform at the time of testing. All testing phases were conducted with no retained session context. Prior chat history and memory were cleared between prompting conditions, such that each transcript was rated in a new session without carryover context.

Structured prompts were developed and saved in Word documents to guide the model’s application of the EXP, as described in the Models and Prompting Protocols section. These prompts were copied from the word documents manually and pasted into the ChatGPT application, and model outputs were copied into a spreadsheet after each response.

1. **Initial Testing Phase**

The initial testing phase used ChatGPT’s flagship model, GPT-4o, to evaluate whether 0-shot, 3-shot, or 7-shot learning produced the most reliable results according to ICC. These prompting conditions were selected in accordance with the aforementioned rationale of comparing 0-shot to few-shot learning, while also accounting for practical considerations and limitations of ChatGPT.

In the first round of testing, all 79 transcripts were rated using only the EXP scale definitions, with no examples provided (0-shot). This process was then repeated in two subsequent iterations. In each new round, ChatGPT was reset (i.e. all prior memory and data were cleared), and the prompt was modified to include examples of transcripts it had rated the most unreliably in the previous round (i.e. those with the largest discrepancies from expert scores)—three examples in the second iteration (3-shot), and seven in the third (7-shot). This iterative procedure was used to initially assess how few-shot learning interacts with performance.

2. **Model Comparison Phase**

The configuration selected from the Initial Testing phase—characterised by relatively higher observed ICCs and fewer exemplars—was applied across four models to assess performance. Each model was provided with the same three structured sets of prompts, and EXP mode and peak scores were collected for all 79 transcripts. No iterative procedures were employed; instead, a fixed set of exemplars derived from the Initial Testing phase was applied consistently across all models.

3. **Protocol Testing Phase**

The model selected from the Model Comparison phase—characterised by relatively higher observed ICCs and favourable efficiency–performance balance (i.e. achieving comparable performance with fewer prompt-based resources)—was further tested using 0-shot, 3-shot, 5-shot, 7-shot, and 10-shot protocols to assess how few-shot learning interacts with performance. Each protocol used the same three structured sets of prompts as the prior phases, and EXP mode and peak scores were collected for all 79 transcripts. Each protocol was treated as an independent inference by resetting the model (i.e. all prior memory and data were cleared). Like the Initial Testing phase, transcripts that were misrated were iteratively added as additional prompt examples.

## Results

**Figure 1.**
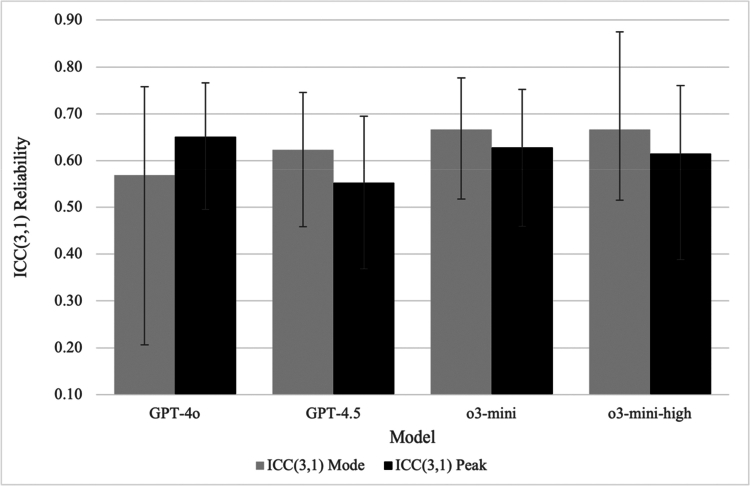
Agreement with manual EXP ratings across models (ICC[3,1]). Note: ICC values are intraclass correlation coefficients, ICC(3,1) (two-way mixed-effects, absolute agreement, single-measures). Conditions reflect models evaluated under a 7-shot prompting protocol. MAD values and F statistics are reported in Table 1. Error bars indicate 95% confidence intervals.

**Figure 2.**
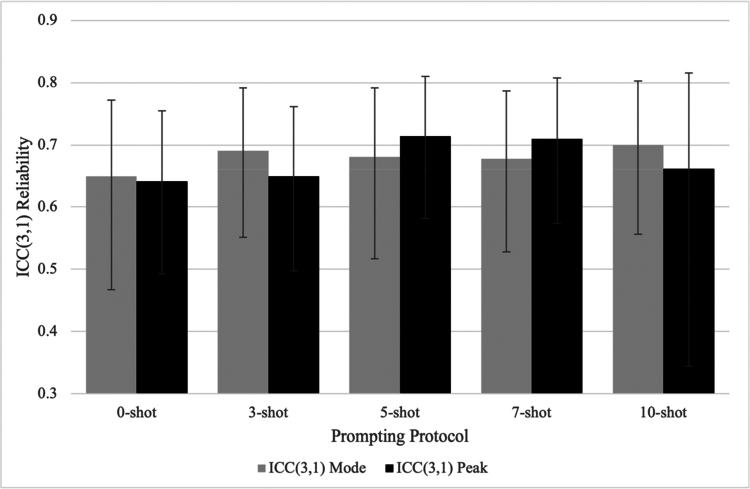
Agreement with manual EXP ratings across prompting protocols for ChatGPT o3-mini (ICC[3,1]). Note: ICC values are intraclass correlation coefficients, ICC(3,1) (two-way mixed-effects, absolute agreement, single-measures). Shot count indicates the number of transcript exemplars included as in-context examples in the prompt. MAD values and F statistics are reported in Table 1. Error bars indicate 95% confidence intervals.

Table 1 summarises agreement with manual EXP ratings across phases, models, and prompting conditions, including ICCs with 95% confidence intervals, associated F statistics, and MAD values for both Mode and Peak scoring. Figures 1 and 2 provide complementary visual summaries of ICC patterns across prompting models and protocols, respectively. Supplemental Figure S1 presents the distribution of signed per-transcript errors (Δ = LLM − Expert) for the o3-mini 5-shot condition measuring Peak EXP.

**Table 1.** Agreement with manual EXP ratings across phases, models, and prompting conditions.

Phase	Model	Shots	ICC Mode (95% CI)	F(df1, df2) Mode	MAD Mode	ICC Peak (95% CI)	F(df1, df2) Peak	MAD Peak
Initial	GPT-4o	0	.55 [.38, .69]	F(78,78) = 3.60	0.99	.50 [.30, .65]	F(78,78) = 3.25	1.00
Initial	GPT-4o	3	.47 [–.04, .73]	F(75,75) = 4.82	1.25	.39 [–.04, .66]	F(75,75) = 3.66	1.29
Initial	GPT-4o	7	.77 [.65, .85]	F(71,71) = 7.59	0.63	.75 [.63, .84]	F(71,71) = 7.38	0.57
Model	GPT-4o	7	.57 [.21, .76]	F(71,71) = 4.94	0.89	.65 [.50, .77]	F(71,71) = 5.00	1.19
Model	GPT-4.5	7	.62 [.46, .75]	F(71,71) = 4.26	0.86	.55 [.37, .70]	F(71,71) = 3.44	0.79
Model	o3-mini	7	.67 [.52, .78]	F(71,71) = 5.04	0.79	.63 [.46, .75]	F(71,71) = 4.64	0.78
Model	o3-mini-high	7	.67 [.52, .88]	F(71,71) = 4.96	0.74	.62 [.39, .76]	F(71,71) = 4.90	0.80
Protocol	o3-mini	0	.65 [.47, .77]	F(78,78) = 5.26	0.94	.64 [.49, .76]	F(78,78) = 4.78	0.82
Protocol	o3-mini	3	.69 [.55, .79]	F(75,75) = 5.41	0.84	.65 [.50, .76]	F(75,75) = 4.66	0.78
Protocol	o3-mini	5	.68 [.52, .79]	F(73,73) = 5.75	0.78	.71 [.58, .81]	F(73,73) = 5.94	0.69
Protocol	o3-mini	7	.68 [.53, .79]	F(71,71) = 5.45	0.86	.71 [.57, .81]	F(71,71) = 5.99	0.75
Protocol	o3-mini	10	.70 [.56, .80]	F(68,68) = 5.62	0.75	.66 [.34, .82]	F(68,68) = 6.52	0.74

Note: ICC values are intraclass correlation coefficients, ICC(3,1) (two-way mixed-effects, absolute agreement, single-measures), with 95% confidence intervals in brackets. F statistics are reported with degrees of freedom. MAD represents mean absolute differences from manual reference ratings. All ICCs were computed in SPSS. Shot count indicates the number of transcript exemplars included as in-context examples.

### Initial testing

The first phase evaluated the impact of few-shot learning on the performance of ChatGPT-4o, and three prompting conditions were tested: 0-shot, 3-shot, and 7-shot learning. ICC(3,1) values for Mode scores were .55 (0-shot), .47 (3-shot), and .77 (7-shot), while Peak scores were .50 (0-shot), .39 (3-shot), and .75 (7-shot). MAD values varied across conditions, with relatively lower values observed in the 7-shot condition (Mode = 0.63; Peak = 0.57), compared to the 0-shot (Mode = 0.99; Peak = 1.00) and 3-shot (Mode = 1.25; Peak = 1.29) conditions.

### Model testing

In the second phase of testing, the 7-shot prompting protocol was applied across four different ChatGPT models: GPT-4o, GPT-4.5, o3-mini, and o3-mini-high. Results suggested some advantage for the reasoning models on Mode scoring, though confidence intervals overlapped across models and differences were less consistent for Peak scores and MAD values. The o3-mini model achieved moderate reliability for Mode (ICC = .67) and moderate reliability for Peak (ICC = .63), with relatively low MAD values across both scoring types (Mode = 0.79; Peak = 0.78). The o3-mini-high model showed broadly comparable performance across Mode (ICC = .67; MAD = 0.74) and Peak (ICC = .62; MAD = 0.80). In contrast, the GPT-4o model showed comparatively lower Mode reliability (ICC = .57) and higher Peak MAD (1.19), while its Peak reliability (ICC = .65) was relatively higher to other models.

### Protocol testing

The final testing phase assessed the effects of few-shot prompting protocols using the o3-mini model, selected from Phase 2 based on its efficiency–performance trade-off. Five prompting protocols were tested: 0-shot, 3-shot, 5-shot, 7-shot, and 10-shot. Results indicated that few-shot prompting generally improved agreement with manual reference ratings and reduced scoring error compared to 0-shot prompting. The 5-shot protocol yielded one of the higher observed reliability estimates (ICC = .68 for Mode, .71 for Peak) and relatively low overall error (MAD = 0.78 for Mode; 0.69 for Peak), though confidence intervals overlapped across conditions. The 7-shot configuration produced comparable results. Although the 10-shot condition yielded slightly higher Mode reliability (ICC = .70), its Peak ICC (.66) was lower than that of the 5-shot configuration and was accompanied by a large confidence interval (95% CI [.34, .82]).

To characterise error patterns in the selected condition, Supplemental Figure S1 presents the distribution of signed per-transcript errors (Δ = LLM − Expert) for Peak EXP scores for the o3-mini 5-shot protocol. Across protocol testing conditions, misratings appeared to be distributed across many transcripts rather than being confined to a small subset; however, we did not conduct formal quantitative analyses to identify transcripts with disproportionately large or frequent errors, nor qualitative analyses to examine the sources of these errors.

## Discussion

Overall, findings suggest that ChatGPT can achieve moderate agreement with manual reference ratings (e.g. ICC between 0.50 and 0.75; Koo and Li (2016) when compared to expert human raters. Specifically, the o3-mini 5-shot condition appeared to offer a favourable efficiency–performance balance, yielding moderate reliability for Mode (ICC[3,1] = .68, 95% CI [.52, .79], *p* < .001) and Peak (ICC[3,1] = .71, 95% CI [.58, .81], *p* < .001). These results should be interpreted with caution, as reliability may be influenced by potential pretraining exposure and is corpus-dependent, and comparisons between ChatGPT-generated ICCs and human reliability benchmarks from other studies are limited. Importantly, these results do not constitute evidence of independent criterion validity. While this level of agreement does not justify replacing human coders, it provides preliminary evidence that LLMs have the potential to serve as a tool to supplement human coding.

Agreement with manual reference ratings was generally moderate across models, with one condition falling below this range. Among them, the o3-mini 5-shot condition yielded comparatively strong ICC estimates across both Mode and Peak EXP scores along with relatively low MAD (see Table 1), though confidence intervals overlapped across conditions. The o3-mini-high model performed comparably but is more resource-intensive. Accordingly, o3-mini was considered the more favourable model in terms of efficiency-performance trade-off.

Differences in observed reliability for the same model across phases should be interpreted cautiously (e.g. the discrepancy between 7-shot prompting for GPT-4o in the Initial Testing and Model Comparison phases). The Initial Testing phase used iterative, performance-informed exemplar selection, whereas the Model Comparison phase used a fixed exemplar set, which likely contributed to this discrepancy. Additional variation may reflect phase-to-phase differences in the ChatGPT product environment across testing dates, as well as run-to-run variability under nominally identical prompting conditions, consistent with the stochastic nature of LLM generation (Herrera-Poyatos et al., 2025).

Across model comparisons, few-shot learning generally outperformed zero-shot prompting. Performance appeared to plateau or slightly decline beyond 5- and 7-shot prompting, suggesting possible diminishing returns with additional examples, potentially due to context window constraints. This pattern is consistent with prior findings that more prompt examples may not always lead to favourable performance for coding processes in LLMs (Brown et al., 2020; Liu et al., 2023). In addition, using more prompts requires greater effort on the part of the researcher. Thus, within the context of the present application, 5-shot prompting may offer a balance between performance and efficiency, particularly in light of token limitations and response costs.

### Strengths

To the author’s knowledge, this study was the first of its kind in using ChatGPT to code the EXP. One of the primary strengths of using ChatGPT or similar LLMs lies in their accessibility, simplicity, and cost-effectiveness. Traditional process coding requires extensive rater training and time investment and is subject to human challenges such as observer drift and fatigue effects (Girard & Cohn, 2016), which limits scalability for research and clinical applications. In contrast, automated coding with LLMs can be performed with minimal overhead, enabling the efficient analysis of larger datasets while maintaining consistent scoring standards.

From a research perspective, it enables the scaling up of process research by allowing efficient analysis of large psychotherapy datasets—something previously constrained by the labour-intensive nature of manual coding. In training and clinical contexts, automated coding tools can support trainees and clinicians by providing automatic feedback on client engagement, offering a valuable supplement to intuition.

### Relation to prior LLM-based psychotherapy transcript coding

The present findings contribute to a growing literature applying LLMs to psychotherapy transcript coding. This includes prior work on Motivational Interviewing (MI) behaviours and microcounseling skills (Ali et al., 2025; Hammerfald et al., 2025), emotion detection in session dialogue (Lalk et al., 2025), patient engagement and process indicators (Eberhardt et al., 2025), and diagnostic classification from counselling transcripts (Sun et al., 2024). Among these, LLM-based coding of MI behaviours and microcounseling skills represents the closest methodological parallels to the present study, as they similarly apply manualized coding frameworks to transcripts. However, these adjacent tasks primarily involve categorical classification of discrete, observable behaviours, whereas EXP coding requires more granular ordinal judgements of clients’ depth of emotional experiencing along a 1–7 continuum, together with adherence to explicit decision rules for aggregating Mode and Peak ratings within segments.

From this perspective, the present findings suggest that LLMs have the potential to code tightly constrained, process-oriented tasks when provided with manual-based, structured prompts. EXP coding likely places greater demands on contextual semantic integration than adjacent skills- or category-based coding tasks. At the same time, the specific nature of the EXP scale may support in-context learning relative to more open-ended constructs such as engagement or affect categories.

### Validity threats: pretraining exposure

Because the evaluation corpus was drawn from a publicly available training manual, it is possible that portions of the EXP manual or related materials were included in the pretraining data of the language models evaluated. If so, observed agreement with expert reference ratings may partially reflect familiarity with manual-derived exemplars rather than generalisable inference from EXP definitions alone. This threat does not invalidate the present proof-of-concept findings but constrains their interpretation. Accordingly, the reported ICCs are best understood as indexing agreement with manual reference ratings under specified prompting conditions, rather than as evidence of criterion validity or out-of-sample generalisation. Establishing the latter will require evaluation on datasets not derived from the EXP manual, a critical next step for future work.

### Ethical and practical feasibility

As noted earlier, the risks associated with cloud-based proprietary services are of particular concern, especially when working with psychotherapy transcripts where re-identification remains a critical issue. This underscores the need for rigorous de-identification protocols and, in some cases, patient consent in future studies. At present, there is no accessible option to download or locally host a stable version of ChatGPT for offline use; all interactions must be conducted through OpenAI’s servers. Alternative open-source large language models such as Meta’s LLaMA can be hosted locally, and researchers have already begun exploring their application in clinical and research settings (Eberhardt et al., 2025). Developing the capacity to host a local, offline LLM represents an essential next step for this line of research, as it would help resolve key ethical concerns (e.g. data privacy and regulatory compliance) and practical challenges (e.g. reproducibility and cost). As it stands, the present study is a proof-of-concept that may be applied either to de-identified data with minimal sensitivity regarding privacy, or to data processed through a securely hosted, local version of an LLM such as LLaMA.

### Limitations

Besides ethical and practical feasibility, several limitations should be acknowledged. First, this study relied on person-centred transcripts from the EXP manual applied to ChatGPT, which may not capture the variability of real-world clinical data; thus, generalisation to real-world psychotherapy transcripts remains untested. Performance may differ across psychotherapy modalities and LLM models or when applied to other process measures with different coding frameworks. Future work should test this approach on diverse datasets and explore its generalisability across models, platforms, and methodologies.

Second, the use of a proprietary, cloud-based model introduces challenges related to reproducibility and transparency. Aside from privacy concerns, ChatGPT’s reliability and performance are influenced by a range of factors, including software updates and fluctuations in computational resources during periods of high user demand (Chen et al., 2023). These variables can lead to inconsistencies in output, which poses challenges for research replication and standardisation—an issue we encountered in our own testing. Specifically, we observed run-to-run variability under nominally identical prompting conditions, reflecting the stochastic nature of LLM generation (Herrera-Poyatos et al., 2025). During exploratory phases of development, repeated runs were conducted to probe model behaviour; however, these exploratory iterations were not logged in a systematic manner.

Some of these issues can be mitigated through methods such as using the OpenAI Application Programming Interface (API; e.g. setting the seed value to lessen randomisation) or through statistical strategies (e.g. running the coding process multiple times to identify false positives). Nonetheless, there remains an inherent risk of unpredictability due to the nature of LLMs as well as the reliance on a third-party provider that requires further investigation and validation.

Finally, the risk of hallucination remains. Xu et al. (2024) demonstrated that hallucination is an inherent and unavoidable feature of all computable large language models, regardless of improvements in architecture, training algorithms, or prompting strategies. In the present study, it is difficult to definitively tell when and if hallucinations occurred, as hallucinations can still result in a correct score. For this reason, we adopted a primarily quantitative perspective, focusing on interrater reliability (i.e. ICC values). In real-life applications where each score’s accuracy and reliability is critical—and until this technology improves—researchers may want to run automated coding multiple times to detect outliers. Another conservative approach is to use ChatGPT as a secondary rater in addition to a human rater.

### Future directions

An important next step is to evaluate model performance on datasets not derived from the EXP manual, ideally using independently collected transcripts with expert ratings. Such out-of-manual validation is necessary to assess generalisability beyond manual exemplars and to more clearly elucidate the extent of potential pretraining exposure effects. These validations should also consider the use of independently collected psychotherapy transcripts that vary in modality (e.g. CBT, psychodynamic, EFT), setting (outpatient, inpatient, telehealth), and transcript quality, including ‘messier’ naturalistic data with disfluencies, overlapping speech, and speech-recognition related errors. Evaluations should compare locally hosted, privacy-favoring models (e.g. LLaMA) with cloud-based systems (e.g. ChatGPT, Gemini, Claude) under consistent prompting, while implementing appropriate safeguards (de-identification, secure storage, and minimal-text sharing) to address privacy and governance concerns. We note that validation on independent datasets is currently underway. Similar approaches should also be applied to other transcript-based process measures. We also note that more granular transcript-level error profiling represents an important direction for future work.

### Prompting and beyond

Since the completion of this study, the literature on systematic prompt design and evaluation has continued to mature. Future work should consider incorporating more formalised strategies, including preregistration of prompt protocols, systematic ablation of prompt components (e.g. instruction hierarchy, exemplars, and format constraints), and robustness analyses across paraphrased prompts to assess surface-level word phrasing sensitivity. These methods may help standardise LLM-based coding approaches and reduce risks of prompt overfitting to specific datasets or formulations.

In addition to prompt-based instruction, several alternative or complementary avenues of research warrant consideration in future work. For example, retrieval-augmented generation (RAG) could dynamically supply EXP definitions, decision rules, and exemplars at inference time. This may have the potential to standardise contextual information, improving consistency across runs.

Fine-tuning on EXP data may also improve consistency and rule adherence beyond prompting alone. However, our preliminary fine-tuning experiments, conducted prior to adopting the present prompt-based methodology, yielded unstable performance, motivating a shift toward the approach used in this study. Although the reasons for this instability are not yet clear, it is plausible that limited dataset size contributed to suboptimal fine-tuning performance. In addition, task characteristics, such as the granularity of the EXP scale and the need for nuanced contextual integration, may also pose challenges for supervised fine-tuning in this domain.

Finally, multi-agent systems, in which multiple independent codings are generated and aggregated via consensus, would likely improve robustness. For example, complementary agents could run multiple passes to identify potential hallucinations, with aggregation rules used to reach a consensus score. Systematic investigation of these paradigms, alongside the present prompting-based approaches, represents an important direction for future methodological research on the EXP and other LLM-based psychotherapy process coding.

## Supplementary Material

Supplementary Material EXP Supplemental Online Materials 2.docx

## Data Availability

The dataset used in this study was derived from transcripts contained in the EXP manual and is subject to copyright and licensing restrictions. Accordingly, it cannot be shared in full. Selected materials included in the Supplementary Materials are provided in a limited, illustrative capacity for transparency and methodological clarity. Readers are referred to the original EXP manual for complete materials. Information on obtaining the EXP manual is available through The International Focusing Institute. Additional data, including analysis code, prompting materials, and derived outputs (e.g. model-generated ratings), may be made available upon reasonable request to the corresponding author.

## Impact statement

This study provides preliminary evidence that large language models can approximate expert ratings on a widely used psychotherapy process measure under certain conditions. In this study, ChatGPT demonstrated moderate agreement with manual reference ratings under specific prompting conditions, suggesting a potential role for AI-assisted coding in psychotherapy research. These findings point to a comparatively favorable efficiency–performance trade-off and may help reduce the time and cost associated with manual coding, while underscoring the need for careful validation and ethical safeguards.

## Clinical or methodological significance of this article

This study introduces a proof-of-concept for automating the coding of a psychotherapy process measure using large language models. By demonstrating preliminary evidence of moderate agreement with manual reference ratings (i.e. ICC between 0.5 and 0.75 based on Koo & Li, 2016), it takes the first step in integrating AI-assisted tools into psychotherapy process research, potentially reducing the time and cost associated with manual coding.
