# Supplementary material for: Exploring the application of large language models in coding the experiencing scale (EXP)
Source: Cogent Ment Health. 2026 Apr 23;5(1):2664163. doi: 10.1080/28324765.2026.2664163 (PMC13107987; doi:10.1080/28324765.2026.2664163)
Supplement: Supplementary Material — EXP Supplemental Online Materials 2.docx [file OAMH_A_2664163_SM6505.docx]

**Supplemental Online Materials**

*Note. Transcript examples from the Experiencing Scale Manual (EXP) included in the prompts have been abbreviated for copyright compliance while preserving the structure and reasoning of the original prompting protocol. EXP scale descriptions and stage language were also paraphrased, consistent with the prompt development process described in the Method section.*

**Reproducibility Checklist**

**Interface and Settings**

*Interface.* ChatGPT macOS application (ChatGPT Plus account)

*Settings.* Customize ChatGPT: Off; Improve the model for everyone: Off; Memory: On

**Testing Phases and Dates**

*Initial Testing.* January 22, 2025 (ChatGPT-4o)

*Model Testing.* March 25, 2025 (ChatGPT-4o, ChatGPT-4.5, o3-mini, o3-mini-high)

*Protocol Testing.* March 30, 2025 (o3-mini)

**Prompt Order**

1. Scale definition prompt
2. Few-shot example prompts
3. Coding instruction prompts
4. Manual transcripts presented in fixed order (A1–H10)

**Session Procedure**

Single predefined evaluation run per condition. Each protocol was evaluated in a fresh session, with prior chats cleared to minimize carryover effects. Model responses were recorded immediately after each run and archived for analysis.

**First Prompt: Scale Definition Prompt**

Use the following reference to identify and score statements or segments on the EXP (Experiencing) scale. Match the content to the highest applicable stage, based on the depth of self-reference, emotional awareness, and internal processing.

Stage 1 – Impersonal, Detached

- Key traits: No personal involvement; speaker is a passive observer or gives generic commentary.
- Language cues: Third-person focus, first-person used only superficially (“I saw,” “I read”).
- Emotion: Absent or mechanical; speaker does not share reactions or feelings.

Examples:

- “I put the lid on the box.”
- “He stepped on my toe.”
- “I read a book about…”

Stage 2 – Personal Involvement, No Emotional Insight

- Key traits: Speaker is personally involved in the situation but doesn’t explore or share inner feelings.
- Language cues: First-person is used explicitly but remains focused on events or roles.
- Emotion: Either missing or treated abstractly/intellectually (“I feel people should be nicer”).

Examples:

- “I wanted to get it done quickly.”
- “I feel that I’m a good worker” (but no elaboration on what that feels like).
- “It was important to me” (without explaining why).

Stage 3 – Emotion Noted, Tied to Specific Events

- Key traits: Feelings are expressed but only in context of a specific situation or role.
- Language cues: Parenthetical emotional comments, retrospective feelings.
- Emotion: Mentioned or described in behavioral or situational terms.

Examples:

- “He didn’t call me back, and I was angry.”
- “It reminded me of being scolded as a child.”
- “When I get mad, I usually leave the room.”

Stage 4 – Inner Experience, Internal Perspective

- Key traits: Clear self-description of feelings and internal states; content centers on experience, not just events.
- Language cues: Rich detail on inner life, self-image, or emotional patterns.
- Emotion: Personal and direct; feelings are the focus rather than background.

Examples:

- “I often feel invisible, like I don’t matter.”
- “I realize I try to please others too much because I fear rejection.”

Stage 5 – Personal Exploration and Hypothesis Testing

- Key traits: Speaker poses a problem or question about their emotional life and explores it meaningfully.
- Structure: (1) Clearly stated emotional/self-related proposition + (2) inward-focused elaboration.
- Emotion: Explored in terms of origin, implications, or conflict.

Examples:

- “Why am I so angry when I feel criticized?”
- “Do I really feel jealous, or is it something else?”
- “I think I get defensive because I’m scared of being wrong.”

Stage 6 – Synthesis and Resolution

- Key traits: The speaker synthesizes emotional insights, leading to a shift or resolution.
- Structure: Immediate emotional insight + new understanding or transformation.
- Emotion: Fully integrated into self-understanding.

Examples:

- “Now I see that my guilt comes from how I was raised to never make mistakes.”
- “I realize I can be angry and still be in control—that changes how I see myself.”

Stage 7 – Expansive, Transformational Insight

- Key traits: Deep self-awareness across multiple domains; insights build on each other.
- Structure: Insights are generalized, applied, reintegrated, or used to form broader self-concepts.
- Emotion: Real-time exploration and transformation; often euphoric or flowing.

Examples:

- “I used to think my fear of failure was isolated to work, but now I see it shows up in my relationships too.”
- “This feeling of acceptance is helping me open up more in every area of my life.”

You are analyzing text segments and must assign two types of ratings based on the EXP scale: mode and peak.

- Mode = the overall, general, or average scale level of the segment.
- Peak = the highest EXP scale level reached at any point in the segment.

Use the following rules to distinguish between them:

1. Equal Division Rule:

If a segment is split evenly between two scale stages, assign the higher stage as the peak, and the lower stage as the mode.

1. Majority Rule:

If more than half of the segment is at a higher stage, both the mode and peak are the same (use the higher stage).

1. Thematic Unity Rule:

If higher-stage statements are frequent and consistent, making the entire segment feel elevated, then mode and peak can be the same.

Example: An abstract idea explained using concrete examples still reflects a high-level theme.

1. Range Rule:

If the segment progresses across multiple stages (e.g., Stage 1 → Stage 2 → Stage 3),

then:

- Assign the highest stage as the peak
- Assign the predominant lower stage as the mode

(Note: The mode can be 2 levels below the peak; intermediate stages may or may not be present.)

Apply this logic when analyzing content to determine the most accurate mode and peak ratings.

**Second Prompt: Few-Shot Example Prompts**

Here are a few example transcript segments, with their corresponding EXP Mode, Peak, and Explanation. Use this structure and reasoning style when scoring future segments

I will provide five therapy transcripts with their assigned EXP scores and reasoning in five separate messages. Here is the first one:

Segment B-5, Mode EXP: 1, Peak EXP: 2

S: In so far as how she's been to my brother, now ah he, it's okay with him if she has a cat, so I ah arranged to buy ah a Siamese kitten down here for $20. And I'm going to ah take it up with me and ah spring it on her at a surprise party…I contacted all of her friends that she wanted to see. Pick them up and bring them over, and have my brother get Mother out of the house on some pretense or another.

*[Additional transcript content omitted and abridged for copyright compliance.]*

Reasoning: The segment is primarily a description of his mother's situation rather than his own. While his analysis of her is fairly detailed, it is stage one because his involvement is passed over. Peaks of two occur when the speaker uses personal pronouns to establish his connection with the narrative, when he justifies his actions, describes his intentions, or compares him-self to his mother, saying "l probably withdrew too." Remember that a two rating cannot be given automatically for content that seems intrinsically important to the listener, for example descriptions of family relations. The speaker must make his role very clear. Level three is not reached because his self-references are intentions, or ideas without any Limited to descriptions of acts, explicit reference to feelings. While he indicates his concern about his mother through this narrative, he has not referred to it specifically enough to be three.

**Third Prompt: Coding Instruction Prompt**

You are an expert trained in applying the EXP (Experiencing) Scale, which ranges from 1 (least personal/impersonal) to 7 (highest emotional insight/self-awareness). The detailed EXP scale definitions and representative examples have already been provided in a previous message. Refer explicitly to those guidelines when analyzing content.

Your task is to analyze segments of a therapy transcript. Each segment comprises multiple utterances (turns of speech). For each segment, complete the following steps:

1. Carefully assess and assign an EXP score (1 to 7) to each individual utterance using the provided EXP manual and stage descriptions. Many short utterances such as “OK,” “uhm,” “Yes sir,” “well…,” or “No,” do not count – skip those.
2. From the individual utterance scores, calculate two distinct ratings for each segment:

- Mode: The most frequently occurring EXP score in the segment (the general or predominant stage).
- Peak: The single highest EXP score observed anywhere within the segment (the highest emotional or experiential level reached).

When determining Mode and Peak, strictly follow these scoring rules:

- Equal Division Rule:

O If a segment is evenly divided between two EXP stages, assign the higher stage as Peak and the lower stage as Mode.

- Majority Rule:

O If more than half of a segment’s utterances are at the higher EXP stage, assign that stage as both Mode and Peak.

- Thematic Unity Rule:

O If higher-stage utterances are frequent, consistent, and elevate the entire segment, assign the higher stage as both Mode and Peak.

- Range Rule:

O For segments progressing clearly across multiple EXP stages (e.g., Stage 1 → Stage 2 → Stage 3):

Assign the highest stage as Peak.

Assign the predominant lower stage as Mode.

(Note: Mode can be up to 2 levels below Peak; intermediate stages may or may not appear.)

Provide your final ratings clearly and concisely for each segment (e.g., A-1, A-2…), in this format:

- Segment ID: Mode = X, Peak = Y

Do not include explanations or reasoning; only report the numeric EXP ratings (1–7).

Segment A-1

S: To this? To the procedure? Well.

S: Well, I think it's …Goodness, well, it's about the best thing that's ever happened for me. So far, I mean, look, uh…And I’ve been... Well , I mean, well , I can definitely see now, I can see that it's no use arguing with anyone about the efficacy of therapy. Just as it's not any use argue, arguing with a Catholic. I mean, you just. . . I can see why clinicians say that, that students should have therapy themselves, even if they don't have any particular problem, um, or any they realize. It's something that - it just changes your thought patterns. Or something.

*[Additional transcript content omitted and abridged for copyright compliance.]*

[Segment A-2, A-3, A-4…]

**Figure S1. Distribution of signed per-transcript errors for o3-mini (5-shot, Peak EXP)**

**
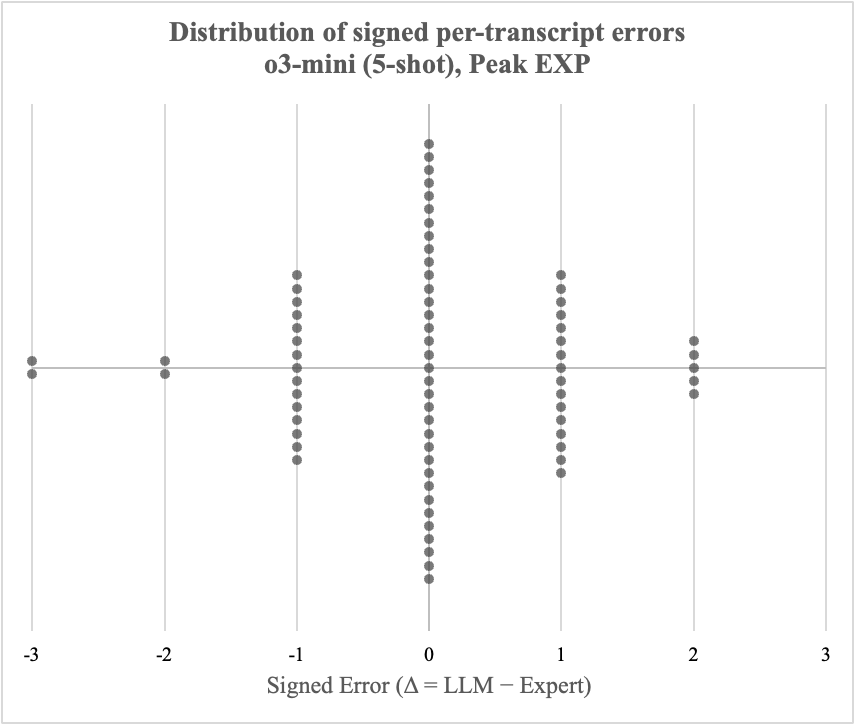
**

*Note*. Each point represents one transcript. Positive values indicate higher model-assigned EXP stages relative to expert ratings, whereas negative values indicate lower model-assigned stages. Points are symmetrically jittered around y = 0 for visualization; vertical position has no substantive meaning and reflects frequency density only.
